# TnBP⁄Triton X-45 Treatment of Plasma for Transfusion Efficiently Inactivates Hepatitis C Virus

**DOI:** 10.1371/journal.pone.0117800

**Published:** 2015-02-06

**Authors:** Ming-Li Chou, Thierry Burnouf, Shun-Pang Chang, Ting-Chun Hung, Chun-Ching Lin, Christopher D. Richardson, Liang-Tzung Lin

**Affiliations:** 1 Graduate Institute of Medical Sciences, College of Medicine, Taipei Medical University, Taipei, Taiwan; 2 Graduate Institute of Biomedical Materials and Tissue Engineering, College of Oral Medicine, Taipei Medical University, Taipei, Taiwan; 3 School of Pharmacy, College of Pharmacy, Kaohsiung Medical University, Kaohsiung, Taiwan; 4 Department of Clinical Pathology, Chi-Mei Medical Center, Tainan, Taiwan; 5 Department of Pediatrics and Canadian Center for Vaccinology, Izaak Walton Killam Health Centre, Halifax, Nova Scotia, Canada; 6 Department of Microbiology and Immunology, School of Medicine, College of Medicine, Taipei Medical University, Taipei, Taiwan

## Abstract

Risk of transmission of hepatitis C virus (HCV) by clinical plasma remains high in countries with a high prevalence of hepatitis C, justifying the implementation of viral inactivation treatments. In this study, we assessed the extent of inactivation of HCV during minipool solvent/detergent (SD; 1% TnBP / 1% Triton X-45) treatment of human plasma. Luciferase-tagged infectious cell culture-derived HCV (HCVcc) particles were used to spike human plasma prior to treatment by SD at 31 ± 0.5°C for 30 min. Samples were taken before and after SD treatment and filtered on a Sep-Pak Plus C18 cartridge to remove the SD agents. Risk of cytotoxicity was assessed by XTT cell viability assay. Viral infectivity was analyzed based on the luciferase signals, 50% tissue culture infectious dose viral titer, and immunofluorescence staining for HCV NS5A protein. Total protein, cholesterol, and triglyceride contents were determined before and after SD treatment and C18 cartridge filtration. Binding analysis, using patient-derived HCV clinical isolates, was also examined to validate the efficacy of the inactivation by SD. SD treatment effectively inactivated HCVcc within 30 min, as demonstrated by the baseline level of reporter signals, total loss of viral infectivity, and absence of viral protein NS5A. SD specifically targeted HCV particles to render them inactive, with essentially no effect on plasma protein content and hemostatic function. More importantly, the efficacy of the SD inactivation method was confirmed against various genotypes of patient-derived HCV clinical isolates and against HCVcc infection of primary human hepatocytes. Therefore, treatment by 1% TnBP / 1% Triton X-45 at 31°C is highly efficient to inactivate HCV in plasma for transfusion, showing its capacity to enhance the safety of therapeutic plasma products. We propose that the methodology used here to study HCV infectivity can be valuable in the validation of viral inactivation and removal processes of human plasma-derived products.

## Introduction

Human blood is a source of important therapeutic products in every country throughout the world, provided that some level of blood collection is in place. Blood products are used to treat various bleeding, immunological, and enzymatic disorders due to congenital or acquired deficiencies, or trauma. Recently the World Health Organization (WHO) has added blood components to its Model List of Essential Medicines [1]. This listing highlights the crucial role that blood products play in human health, and should act as a strong incentive for governments to ensure an adequate supply of safe blood products at the national level. Plasma for transfusion is an important blood component. The recognized indications of plasma for transfusion in developed countries are strictly defined and include: replacement therapy for single coagulation factor deficiencies, if individual factor concentrates are not available; disseminated intravascular coagulation associated with multiple coagulation factor deficiencies; thrombotic thrombocytopenic purpura; liver disease; reversal of warfarin effect associated with bleeding; or surgical bleeding and massive transfusion [2]. In the developing world, the lack of supply in fractionated plasma products leads to prescribing plasma to patients suffering from other pathologies [3,4]. In countries with a challenging epidemiological environment, such as those exposed to known and emerging pathogens while also facing a lack of government support, sufficient safe blood donors, and adequate health regulations, the clinical use of plasma is associated with significant risks of transfusion-transmitted viral infections [5].

A major potential infectious complication of plasma transfusion therapy is the transmission of hepatitis C virus (HCV), a highly pathogenic blood-borne agent that is extremely adept at establishing persistent infection. HCV is a positive-stranded RNA flavivirus with a genome that includes structural (core and glycoproteins E1, E2) and non-structural proteins (p7, NS2, NS3, NS4A, NS4B, NS5A, and NS5B) [6]. Acute and chronic HCV infection is a heavy burden globally, affecting approximately 150–300 million people. Treatment using pegylated interferon-α combined with ribavirin has been the major standard of care (SOC) for hepatitis C for over a decade, yielding limited success for the most widely distributed genotype 1 infections [7]. Recent development of inhibitors targeting the viral NS3/4A protease (Boceprevir, Telaprevir, and Simeprevir), the NS5A cofactor (Daclatasvir, Ledipasvir, and Ombitasvir), and the NS5B polymerase (Sofosbuvir, Mericitabine, and Dasabuvir) has improved the treatment modalities for hepatitis C, with a higher success rate and less adverse reactions and toxicity [8]. However, the high cost associated with these newer treatments makes it difficult to treat every HCV-infected individual, particularly in low resource nations. More importantly, hepatitis C will remain a major health issue worldwide as long as a preventive vaccine against the virus is unavailable. In some countries such as Egypt, HCV infection can affect 10% or more of the population [9,10]. Chronic hepatitis C infection carriers have high risks of developing liver cirrhosis and liver cancer. Approximately 500,000 people die of hepatitis C-related diseases every year [11] and this viral infection is currently among the top indications for liver transplantation in many countries [12,13].

Despite the implementation of screening procedures, HCV transmission associated with blood product transfusion remains an issue in endemic areas and in resource-limited countries due to poorly controlled blood screening practices [14]. Measures to improve the safety of plasma for transfusion encompass the implementation of a viral inactivation treatment sufficiently robust to destroy viral infectivity without affecting the plasma hemostatic power of native coagulation factors. A minipool viral inactivation treatment of plasma by solvent/detergent (SD) has been recently developed in Egypt [15,16]. In this process, plasma is incubated at 31°C with tri-*n*-butyl phosphate (TnBP) as solvent and Triton X-45 as detergent. The efficacy of this treatment in inactivating HCV has not been reported yet due to the difficulty in using an animal model such as chimpanzee, and a lack of an *in vitro* cellular model suitable to assess HCV infectivity prior to the development of recombinant viruses [17–19].

In this study, using infectious cell culture-derived HCV (HCVcc), we show for the first time the capacity of an SD treatment of plasma combining TnBP and Triton X-45 to efficiently inactivate HCV. The efficacy of this minipool SD treatment is also verified against clinical HCV isolates and primary human hepatocyte infection by HCVcc.

## Materials and Methods

### Plasma Samples Collection and Preparation

Plasma donations were collected using a Haemonetics machine (Braintree, MS, USA) in the presence of a citrate anticoagulant solution and following the manufacturer’s instructions, from healthy volunteers who provided verbal informed consent. The procedure was approved by the Institutional Review Board of Taipei Medical University (IRB#201305054) with waiver of documentation of consent. Plasma donations were kept at 20–24°C for up to 6 h before sampling. Under sterile conditions, plasma samples were then dispensed into 1 ml aliquots and frozen immediately at < -20°C until use.

### Reagents, Cell Culture, and Virus Preparation

Dulbecco’s modified Eagle’s medium (DMEM), fetal bovine serum (FBS), gentamycin, and amphotericin B were purchased from GIBCO-Invitrogen (Carlsbad, CA, USA) and dimethylsulfoxide (DMSO) from Sigma (St. Louis, MO, USA).

The SD minipool treatment was prepared using a 1:1 mixture of tri-*n*-butyl phosphate (TnBP; Merck KGaA, Darmstadt, Germany) and Triton X-45 (Sigma).

The Huh-7.5 hepatoma cells (obtained from Dr. Charles M. Rice) were cultured in DMEM supplemented with 10% FBS, 50 μg/ml gentamicin, and 0.5 μg/ml amphotericin B.

Production of HCVcc by electroporation of hepatoma cells using the *Gaussia* luciferase reporter-tagged Jc1FLAG2(p7-nsGluc2A) construct (genotype 2a; kindly provided by Dr. Charles M. Rice) has been described elsewhere [20]. HCV viral titer was determined by 50% tissue culture infectious dose (TCID_50_) according to earlier methods [21] using immunofluorescence staining of NS5A as described below. The basal medium containing 2% FBS with antibiotics was used for all virus infection experiments.

### Study Design

**Fig 1:**
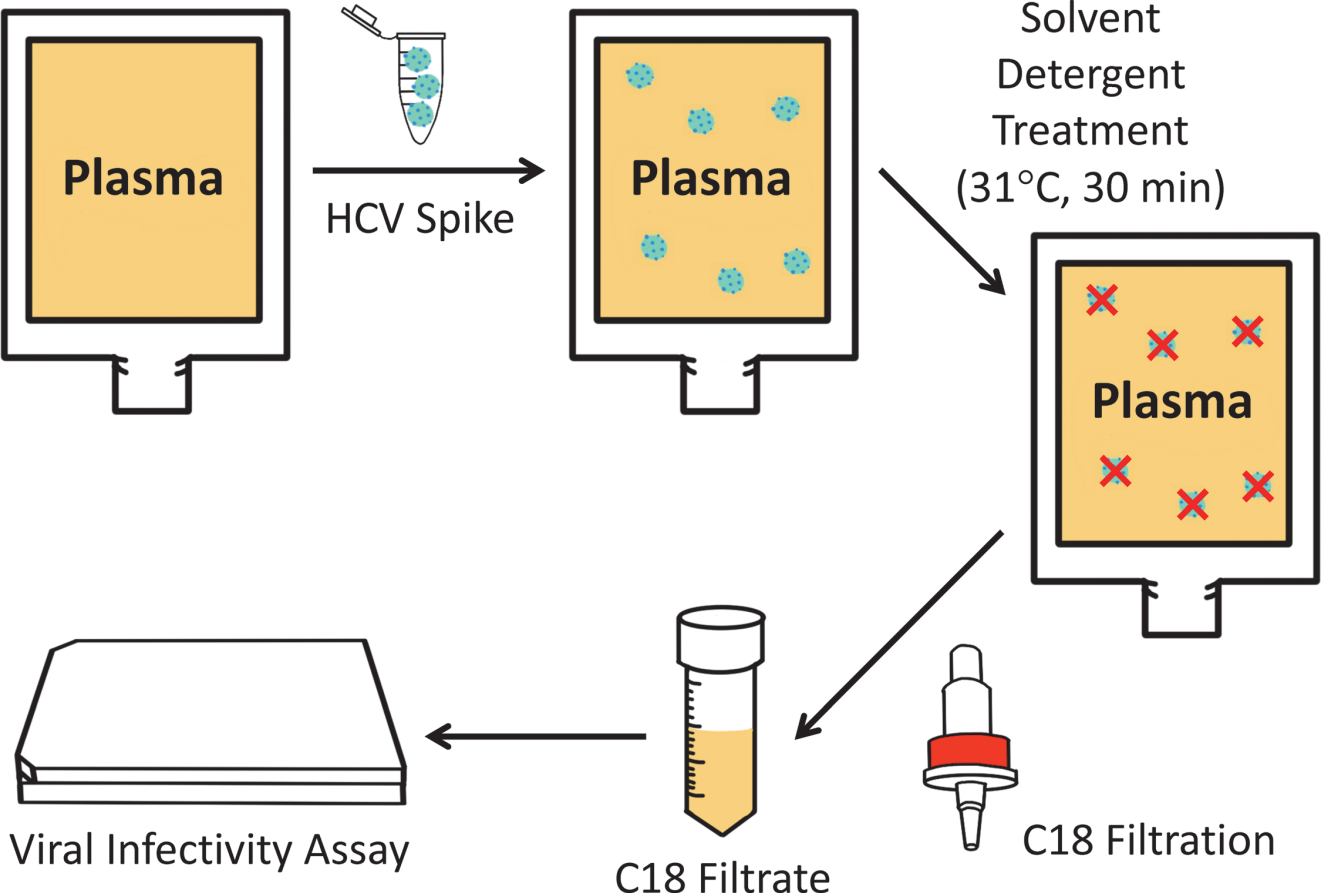
Flowchart of SD treatment and C18 filtration procedure on human plasma for HCV infectivity assay.

Plasma samples (thawed at 31°C) were evaluated for their potential cytotoxicity on Huh-7.5 cells prior to all experiments. Plasma samples were spiked with the HCVcc stock at a ratio of 9:1 (plasma:HCV stock) in accordance with regulatory guidelines and international recommendations [22]. For inactivation by SD, virus-spiked samples were treated with SD agents at a final concentration of 1% (v/v), each followed by vortexing and incubation for 30 min at 31°C under mild mixing. The treatment was stopped by processing the samples (0.3 ml) on Sep-Pak Plus C18 cartridge 55–105 μm cartridges (Waters Corporation, Milford, MA, USA) containing 130 mg of octadecyl (C18) material, which is known to quickly remove the SD agents and stop the reaction [23]. Samples containing plasma only, HCV-spiked plasma, or HCV-spiked plasma treated by SD minipool with or without subsequent C18 cartridge filtration were then evaluated for viral infectivity. A schematic of the procedure is shown in Fig. 1.

### Cytotoxicity Assay

Sub-confluent Huh-7.5 cells (1 × 10^4^ cells/well of 96-well plates) were treated with the following test plasma samples or controls: plasma only (diluted 1:10 in media), phosphate buffered saline (PBS) treated with SD (1% TnBP / 1% Triton X-45), and plasma treated with SD followed by C18 cartridge filtration. After 72 h, cell viability (%) was then assessed using the XTT (2,3-bis[2-methoxy-4-nitro-5-sulfophenyl]-5-phenylamino)-carbonyl]-2H-tetrazolium hydroxide)-based *in vitro* toxicology assay kit (Sigma), as previously reported [24].

### Viral Infectivity Assay

Huh-7.5 cell monolayers (1 × 10^4^ cells/well of 96-well plates) were challenged at 37°C for 3 h with serially diluted samples (HCVcc-spiked plasma [multiplicity of infection, MOI = 0.01] with or without SD treatment and subsequent C18 filtration), followed by removal of excess virion by PBS wash twice before incubation in a medium containing 2% of FBS. After 72 h, the supernatant was collected and then assayed for luciferase activity using the BioLux *Gaussia* Luciferase Assay Kit (New England Biolabs; Pickering, ON, Canada) and a luminometer (Promega; Madison, WI, USA). HCV infectivity was expressed as log_10_ of relative light units (RLU) for determining viral inhibition (%) and calculated using GraphPad Prism 5 software (San Diego, CA, USA) as previously reported [21]. All values were plotted against the control treatment.

### Viral Inactivation Assay Using Low Dose SD

Viral inactivation assay was performed as previously described [21]. Briefly, HCVcc was treated with low dose SD (0.01% or 0.05%) and then incubated at 31°C for 30 min. The virus inoculum was subsequently diluted 50-fold with 2% FBS medium to achieve non-cytotoxic concentrations of SD agents (≤ 0.001%), and then inoculated on the Huh-7.5 cells seeded in 96-well plates (final viral MOI = 0.01). Following 3 h of incubation, wells were washed with PBS twice before applying the overlay medium for another 72 h of incubation. Infection of the monolayer was detected by luciferase assay, as just described.

### Immunofluorescence Staining for Viral Protein

For immunofluorescence analysis of HCV infectivity and TCID_50_ [21], the wells were washed and fixed with ice-cold methanol before blocking with 3% of BSA. Samples were then treated at 37°C for 1 h with the mouse monoclonal anti-NS5A 9E10 antibody (1:25,000; gift from Dr. Charles M. Rice), followed by PBS washes three times before detection using the Alexa Fluor 488 goat anti-mouse IgG (H + L) antibody (Invitrogen; 1:400). Following incubation at 37°C for 1 h, the samples were washed with PBS three times prior to staining the nuclei with Hoechst dye (Sigma) and visualization by fluorescence microscopy. Micrographs were taken from 3 random fields per sample for each independent experiment.

### Biochemical Analysis for Plasma Composition and Evaluation of Plasma Hemostatic Function

Plasma samples were frozen ≤ 20°C for up to one month. On the day of analysis, samples were thawed at 30°C and analyzed within 2 h. C18-filtered and SD/C18-treated plasma samples were tested for total protein, triglycerides, cholesterol, apolipoprotein A1 (ApoA1), and apolipoprotein B (ApoB) by ADVIA 1800 Chemistry System (SIEMENS, Munich, Germany). Prothrombin time (PT) and activated partial thromboplastin time (APTT) were assessed at Taipei Medical University Hospital and Union Clinic Laboratory (Taipei, Taiwan) using routine validated procedures by Sysmex CA-1500 System (SIEMENS) and the international normalized ratio (INR) was determined. Results were compared to normal range values of each analysis.

### HCV Binding Analysis Using Clinical Isolates

To evaluate the efficacy of SD minipool with subsequent C18 cartridge filtration against clinical HCV isolates, a surface binding analysis of serum HCV on hepatoma cells was performed as previously described with some modifications [25]. Virus derived from hepatitis C patients (viral load > 1 x 10^6^ IU/ml; negative for hepatitis A virus [HAV], hepatitis B virus, and human immunodeficiency virus [HIV] markers) were treated with or without SD and C18 before inoculating Huh-7.5 monolayers at 4°C and washing off any unbound virus with PBS. Viral RNA from cell-bound virus was isolated by extracting total cellular RNA using Trizol (Invitrogen) and then subjected to quantitation by COBAS AMPLICOR HCV MONITOR test (Roche Molecular Diagnostics; Pleasanton, CA, USA) according to the manufacturer’s protocol. The use of HCV-positive patient blood samples from the Chi-Mei Medical Center Biobank for research purposes was approved by the Institutional Review Board of Chi-Mei Medical Center.

### HCVcc Infection of Primary Human Hepatocytes

Infection of primary human hepatocytes using HCVcc was based on reported methods [26] with modifications. Primary human hepatocytes obtained from GIBCO-Invitrogen were seeded in collagen I-coated plates and cultured in William’s medium E containing primary hepatocytes supplements (GIBCO-Invitrogen) according to the manufacturer’s instructions. Two days post seeding, the cells were washed with PBS and then inoculated with plasma only or HCVcc-spiked plasma (MOI = 0.1) with or without SD treatment followed by C18 filtration at 37°C for 3 h. Subsequently, the wells were washed with PBS twice to remove excess virion and then overlaid with the primary hepatocyte culturing medium described above. After 5 days of incubation, the supernatant was collected and then assayed for luciferase activity as indicated earlier.

### Statistical Analysis

Statistical analysis was performed by one-way ANOVA followed by Dunnett's multiple comparisons test. A *P* value of < 0.05 was considered as statistically significant. Data were presented as means ± standard errors of the means (SEM) from three independent experiments unless otherwise indicated.

## Results

### Plasma Treated with SD Followed by C18 Filtration Eliminates SD Cytotoxicity

**Fig 2:**
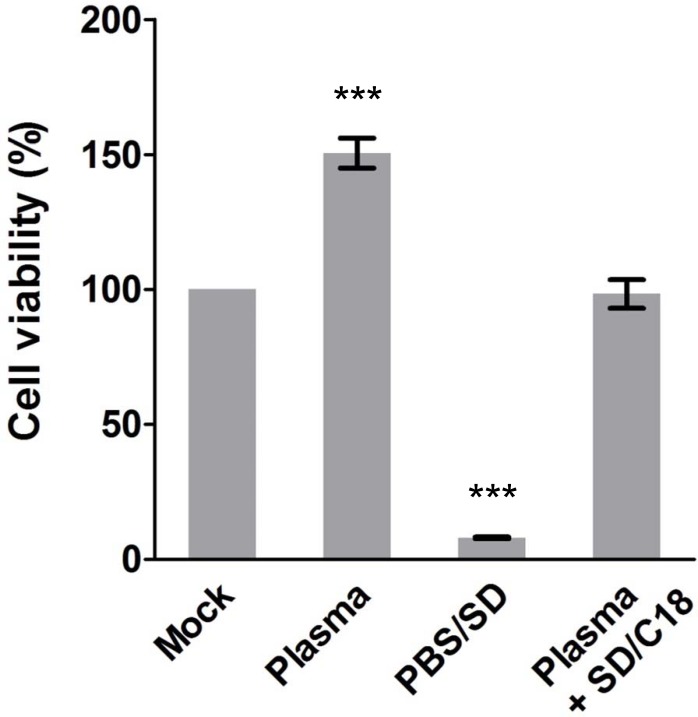
C18 efficiently removes SD cytotoxicity. Plasma and SD/C18 treatments were tested for potential cytotoxicity. Huh-7.5 cells seeded at sub-confluence in 96-well plates (1 × 10^4^ cells per well) were incubated in various treatments for 72 h before analysis by XTT cell viability assay. Data shown are collected from three independent experiments with means ± SEM (***: *P* < 0.001). Plasma: virus-free plasma; PBS/SD: PBS treated with SD (1% TnBP / 1% Triton X-45); Plasma + SD/C18: C18 cartridge filtration of plasma treated with SD.

We first carried out a cell viability assay using XTT to demonstrate that plasma alone and SD-treated plasma followed by C18 filtration (SD/C18) do not induce cytotoxicity. Treatment with plasma only was not cytotoxic to the Huh-7.5 cells, whereas treatment with the minipool SD (1% of TnBP / 1% of Triton X-45) produced, as expected, significant cell death. This cytotoxic effect from SD however could be completely removed by C18 filtration as shown in the SD-treated plasma group with C18 treatment (Fig. 2). Therefore, hydrophobic C18 filtration can efficiently remove SD that is present in the plasma sample. The reduced viability observed when using the SD-treated plasma, compared to untreated plasma, also suggests removal of some growth factors or hydrophobic nutrients during the SD procedure.

### HCV Particles are Inactivated by SD Treatment

**Fig 3:**
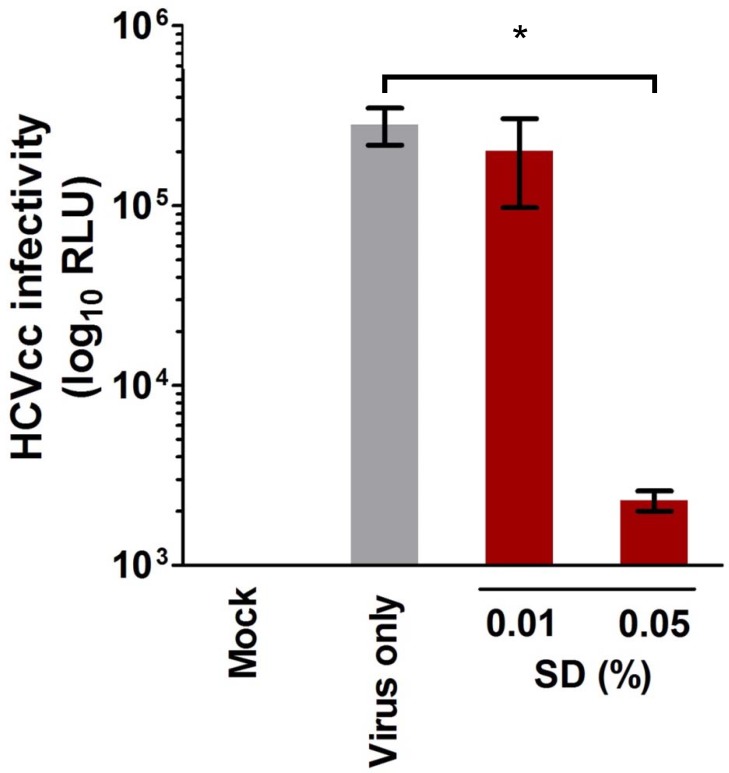
SD directly inactivates HCV particles. To test the specificity of SD in inactivating HCV particles, cell-free virus was co-incubated with low dose SD (0.01%, 0.05%) for 3 h and then diluted 50× to sub-cytotoxic concentrations of the treatment before infecting Huh-7.5 cells (MOI = 0.01). Viral infectivity was measured by luciferase reporter activity (RLU). Data shown are collected from three independent experiments with means ± SEM (*: *P* < 0.05).

To further analyze the specificity of SD in inactivating HCV particles in plasma-free conditions, cell-free virus was co-incubated with only low concentrations of SD (0.01% and 0.05%) at 31°C for 30 min. Following the SD reaction, the mixture was diluted 50-fold to sub-cytotoxic concentration of SD and then used to challenge Huh-7.5 cells (final MOI = 0.01) to test the remaining viral infectivity. Results indicated that using as little as 0.05% of SD (0.025% of TnBP and 0.025% of Triton X-45) reduced reporter signals to near background levels (Fig. 3). This suggests that treatment with a minimum of 0.05% of SD could, in plasma-free conditions, specifically and efficiently inactivate HCV particles to prevent viral infection.

### SD Treatment Efficiently Removes HCV Infectivity in Human Plasma

**Fig 4:**
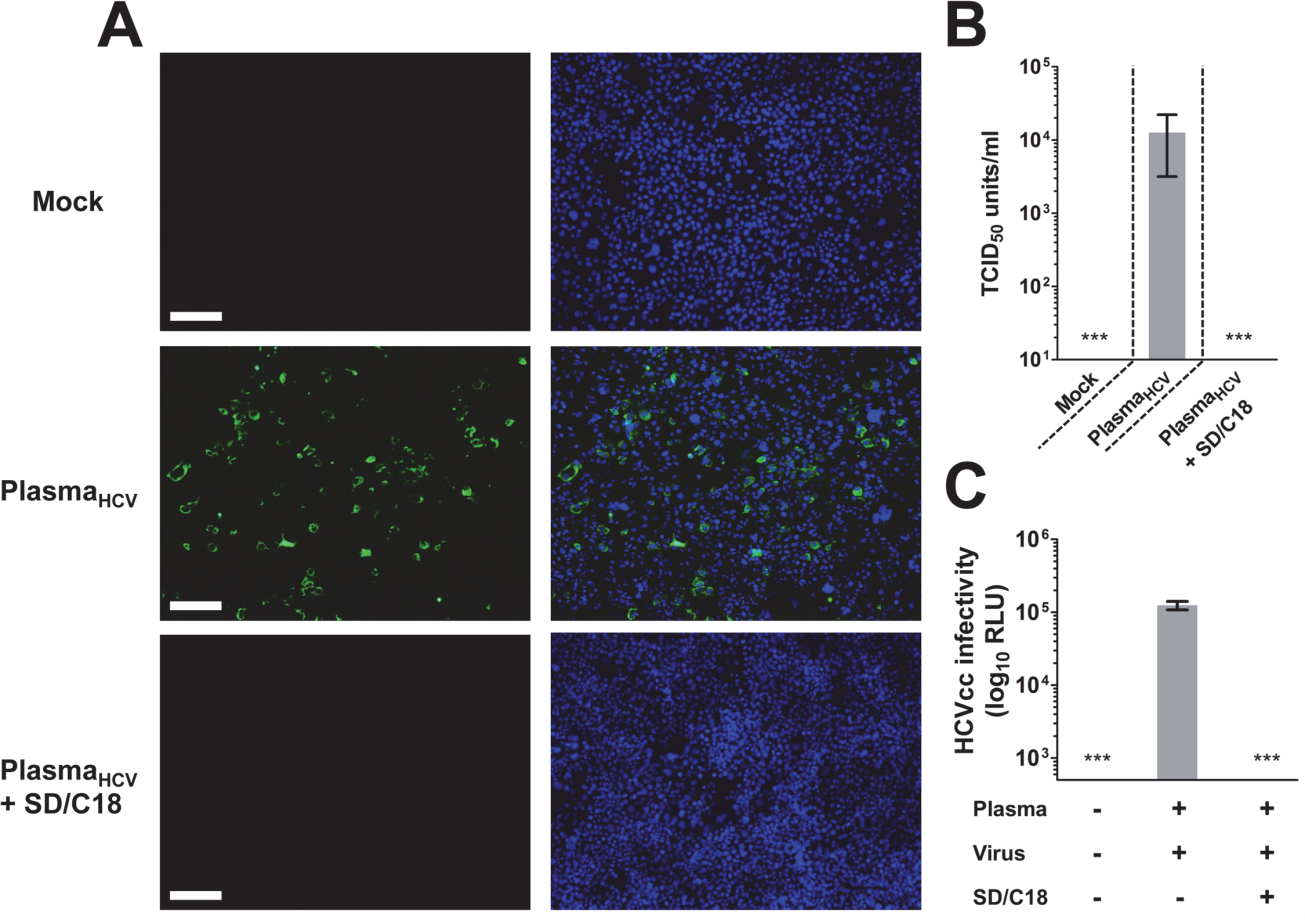
SD treatment efficiently removes HCV infectivity from virus-containing plasma. Infectivity of HCV-spiked plasma (Plasma_HCV_) with or without SD treatment followed by C18 filtration (SD/C18) was analyzed by: **A.** Immunofluorescence staining of viral NS5A (magnification: 100×; scale bar = 100 μm); **B.** Virus titer determination by TCID_50_ analysis (units/ml); and **C.** Luciferase reporter measurement of viral infectivity (RLU). All quantifiable data and representative micrographs are from three independent experiments; error bars indicate means ± SEM (***: *P* < 0.001). See text for details.

To demonstrate the capacity of SD treatment in rendering HCV-containing plasma non-infective, HCV-spiked plasma was treated with SD (1% TnBP / 1% Triton X-45) followed by C18 filtration, before challenging Huh-7.5 cells. The C18-filtered-SD treatment efficiently abolished HCV infectivity as demonstrated by the lack of viral NS5A protein immunostaining compared to the non-treated plasma containing HCV (Fig. 4A), which is indicative of an absence of virus replication. This observation is reflected by the undetectable virus titer in the HCV-spiked plasma sample after the same SD treatment in the TCID_50_ analysis (Fig. 4B). Similarly, luciferase reporter levels from the HCV-spiked plasma infection was completely reduced to baseline following SD/C18 procedure, again suggesting absence of infection (Fig. 4C). Thus, minipool SD treatment can efficiently remove HCV infectivity from plasma.

### SD Treatment Followed by C18 Filtration Does Not Alter Plasma Protein Content and Hemostatic Function

To confirm that the contents and functionality of plasma are preserved following the SD procedure, and to verify the correct scaling-down of the SD treatment as suggested in viral validation guidelines [22], we next investigated the effect of SD and C18 treatments on key biochemical markers of the composition and hemostatic property of human plasma. Levels of total protein, triglyceride, cholesterol, and apolipoproteins (ApoA1 and ApoB) were measured following the treatments and compared with normal physiological values [27,28]. Results indicated that these biochemical indices from plasma treated with SD/C18 or C18 alone are not significantly different from normal range values (Table 1). However, when the treatments were compared to each other, levels of triglyceride, cholesterol, and ApoB in the original plasma were decreased after the SD/C18 treatment without affecting total protein content, in contrast to C18 filtration alone which had not effect (Table 1). This observation is consistent with the notion that SD reaction targets and reduces lipids without affecting plasma protein content [23]. Together, the data suggest that the overall biochemical composition, in particular the total protein level, of human plasma after SD/C18 treatment was generally maintained within or close to the range of normal physiological values, with a tendency to reduce lipid-rich molecules including triglyceride, cholesterol, and ApoB. This is consistent with production batches data and therefore confirms the correct scaling-down of the process.

**Table 1:** Biochemical composition of plasma with SD and C18 treatments.

Parameter	Normal Range	Control	C18	SD + C18	*P* Value
Total Protein (g/dl)	6.0–7.8	6.30 ± 0.11	6.25 ± 0.07	5.80 ± 0.05	NS
Triglyceride (mg/dl)	< 250	96.00 ± 3.78	82.73 ± 11.92	53.33 ± 1.20	NS
Cholesterol (mg/dl)	< 199	139.7 ± 8.7	134.6 ± 15.8	94.3 ± 1.4	NS
ApoA1 (mg/dl)	> 88	104.0 ± 7.9	106.4 ± 7.8	106.7 ± 1.6	NS
ApoB (mg/dl)	< 151	75.00 ± 7.63	78.93 ± 9.17	44.33 ± 0.33	NS

Levels of total protein, triglyceride, cholesterol, apolipoprotein A1 (ApoA1), and apolipoprotein B (ApoB) in plasma are quantitated following SD and C18 treatments. Control: plasma only; C18: plasma with C18 cartridge filtration; SD + C18: plasma treated with SD followed by C18 cartridge filtration. SD treatment is known to reduce lipids without affecting plasma protein content. Data shown are collected from three independent experiments with means ± SEM. NS = not significantly different (*P* > 0.05).

To further ensure that our SD/C18 procedure, as carried out in this study, did not impact the plasma hemostatic function, we performed analyses to determine its global coagulation activity using prothrombin time (PT), international normalized ratio (INR), and activated partial thromboplastin time (APTT) on plasma treated by SD/C18. As shown in Table 2, plasma hemostatic properties were retained in human plasma treated by either C18 alone or by SD/C18, with values of PT, INR, and APTT being maintained within normal range [16,29], confirming the correct implementation of the treatment. Thus, scaling down the SD/C18 procedure does not compromise the hemostatic function of the treated plasma.

**Table 2:** SD treatment followed by C18 filtration does not impact plasma hemostatic function.

Parameter	Normal Range	Control	C18	SD + C18	*P* Value
PT (sec)	11–13.5	11.37 ± 0.06	11.6 ± 0.20	12.73 ± 0.08	NS
INR	0.8–1.2	1.047 ± 0.006	1.067 ± 0.017	1.170 ± 0.005	NS
APTT (sec)	22–41	33.87 ± 0.68	34.57 ± 1.31	34.93 ± 0.06	NS

Retention of plasma bioactivity following SD and C18 treatments was measured for: A. Prothrombin time (PT; sec); B. International normalized ratio (INR); and C. Activated partial thromboplastin time (APTT; sec). Control: plasma only; C18: plasma with C18 cartridge filtration; SD + C18: plasma treated with SD followed by C18 cartridge filtration. Values are means ± SEM from three independent experiments. NS = not significantly different (*P* > 0.05).

### Capacity of SD Treatment to Inactivate HCV is Demonstrated Against Clinical HCV Isolates and Infection of Primary Human Hepatocytes

**Fig 5:**
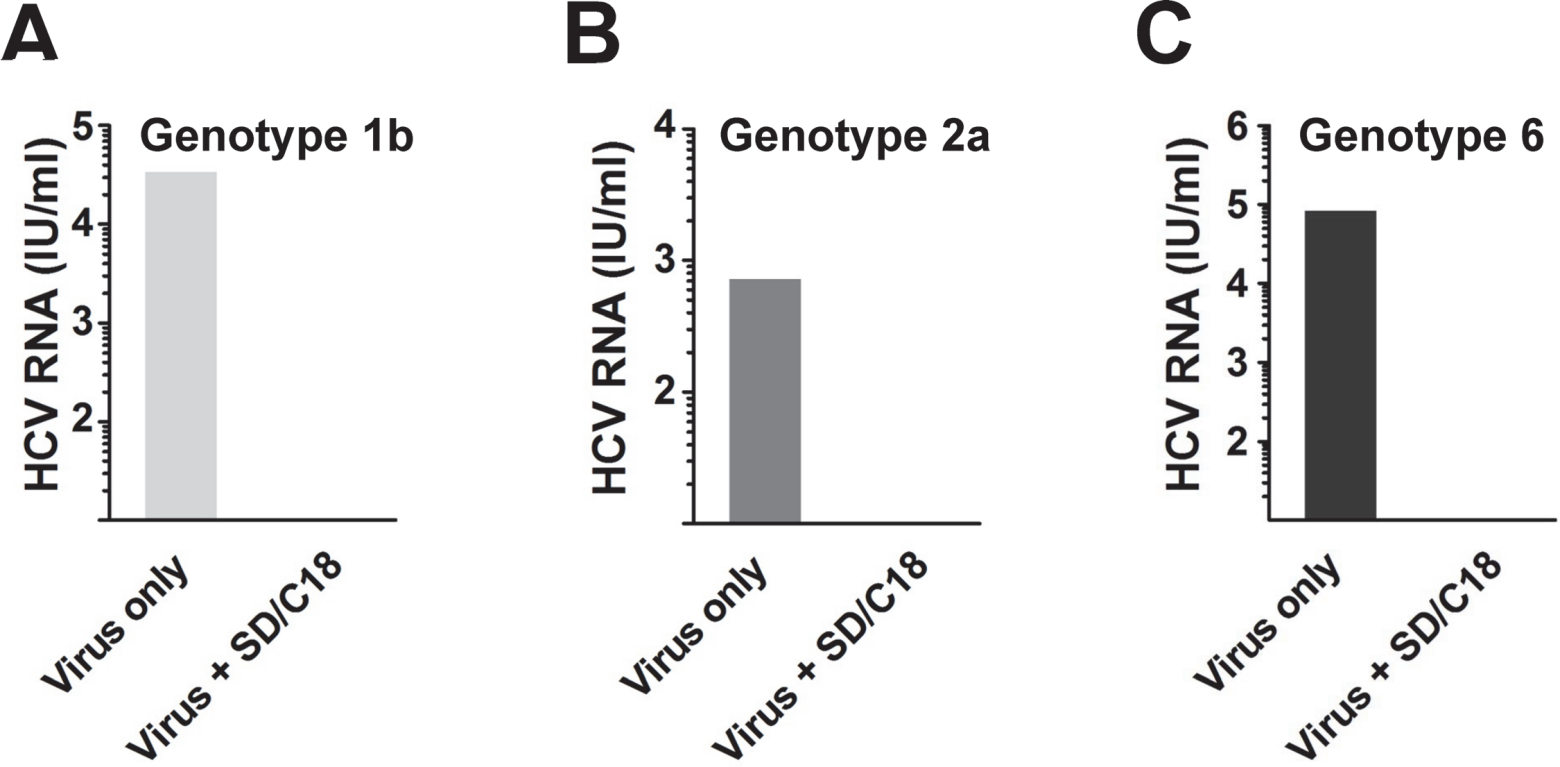
SD treatment efficiently inactivates clinical HCV isolates. Patient-derived HCV particles of various genotypes were treated with or without SD/C18 procedure and then used to challenge Huh-7.5 cells. Total HCV RNA on Huh-7.5 cell surface was subsequently extracted and quantified by COBAS AMPLICOR HCV MONITOR test. Mean values from duplicate experiments are shown (IU/ml).

To further substantiate the SD treatment as a valid method for inactivating HCV in a clinical setting, we tested the minipool procedure against patient-derived HCV particles using a virus binding analysis [25]. Although this particular analysis is not an infectivity assay, it does allow direct examination of the capacity of the SD treatment for inactivating clinical virus particles and preventing their binding onto the hepatoma cells. To this end, clinical HCV isolates (genotypes 1b, 2a, and 6) treated with SD and then filtered by C18 were used to challenge Huh-7.5 cells, and following the washing off of any unbound viral particles, total RNA was extracted to quantitate surface-bound virus. As shown in Fig. 5, patient-derived HCV RNA was undetected in the SD/C18 treatment group compared to viral inoculum without the treatment, suggesting inactivation of the clinical isolates.

**Fig 6:**
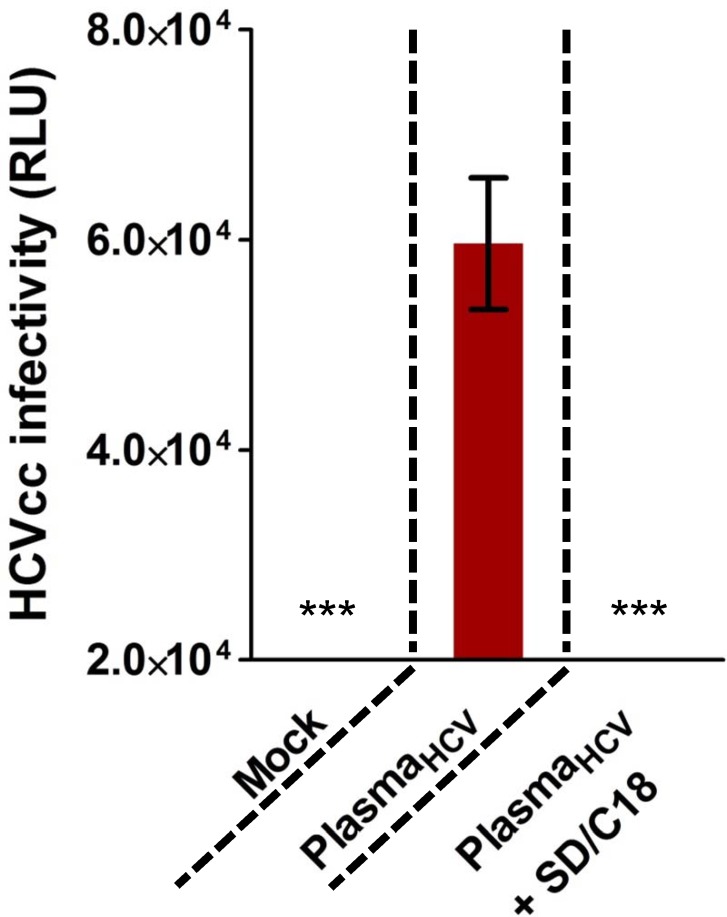
HCVcc infection of primary human hepatocytes is abolished by SD treatment. Plated primary human hepatocytes were challenged with plasmas only (Mock), HCVcc-spiked plasma (Plasma_HCV_), or HCVcc-spiked plasma with SD treatment followed by C18 filtration (Plasma_HCV_ + SD/C18), then washed, and subsequently analyzed for luciferase activity (RLU) in the supernatant to quantify viral infectivity following 5 days of incubation in primary hepatocyte culturing medium. Error bars indicate means ± SEM from three independent experiments (***: *P* < 0.001). See text for details.

In addition, we assessed the capacity of the minipool SD to nullify the HCVcc-spiked plasma inoculum to prevent infection of primary human hepatocytes [26]. Seeded primary human hepatocytes were challenged with plasma only or HCVcc-spiked plasma, with or without SD/C18 treatment, and then measured for luciferase reporter signals after 5 days of incubation. Similar to earlier observed effects, luciferase activity reflecting virus infection was significantly reduced in HCV-spiked plasma treated with SD/C18 compared to the virus plasma control, which is indicative of loss of HCVcc infectivity in the primary human hepatocytes following SD/C18 procedure (Fig. 6).

Altogether, these observations demonstrate the capacity of the minipool SD treatment followed by C18 filtration to inactivate HCV and suggest it as a valid approach for reducing the risk of HCV infection from plasma products in a clinical setting.

## Discussion

Plasma for transfusion is now listed as an essential medicine by the WHO, highlighting its therapeutic value and raising government awareness on the importance of ensuring its availability and safety at a national level [1]. In spite of progress in donor screening and, particularly, donation testing, HCV and other blood-borne viruses can still be transmitted by clinical plasma, with a higher incidence in countries with an underdeveloped blood collection organization and lack of a regular safe blood donor base [30]. Viral transmission can be avoided by using procedures capable of inactivating viruses in plasma without altering its hemostatic properties [22]. 1% TnBP / 1% Triton X-100 SD treatment was the first viral inactivation method implemented on industrial pools of 100–500 plasma donations [23,31]. This procedure inactivates viruses by dissolving their lipid membrane [32]. A drawback of this treatment, otherwise very effective [33], is the fact that Triton X-100 must be removed by large-scale hydrophobic interaction chromatography, which is an expensive and sophisticated process that requires a pharmaceutical facility and is therefore difficult to implement in developing countries. Three viral inactivation methods have been developed for the treatment of single-donor plasma donations: methylene blue/illumination [34], psoralen/ultraviolet light (UV) [35], and ribloflavine/UV [36]. They inactivate viruses by photochemical alteration of nucleic acids [37]. Recently a simplified SD treatment of plasma and cryoprecipitate using 1% TnBP / 1% Triton X-45, as in the present study, has been developed to facilitate its implementation in developing countries [15,16]. This treatment has already been shown to maintain the global hemostatic properties of plasma, and the use of Triton X-45 was shown to preserve better than Triton X-100 the functional activity of individual coagulation factors and protease inhibitors [38]. In addition, this 1% TnBP / 1% Triton X-45 treatment of plasma was formerly validated for its capacity to inactivate several enveloped viruses by duplicate small-scale spiking experiments [39]. The validation was done under worst-case conditions (low range of SD content and at a temperature of 29.5 ± 0.5°C) and with several time-points to assess residual infectivity of three viruses, following the recommendations of the WHO Guidelines [22]. The study demonstrated reduction factors of > 4.17, > 4.73, and > 4.72 for HIV, bovine viral diarrhea virus (BVDV), and pseudorabies virus, respectively, within 2 min of treatment [39]. However, the effect of such inactivation method has not yet been evaluated against HCV directly, which prompted us to perform the present experimental study to confirm the capacity of the SD treatment to inactivate HCV.

One important aspect in the development of any viral inactivation method of plasma is validating its capacity to destroy sufficient doses of infectious agents. In the past, demonstration of virus inactivation efficacy used animal infectivity models, such as chimpanzees, that were exposed to a challenge with infectious material subjected or not to inactivation treatment, and were followed for potential signs of infections [23,40]. Currently, viral inactivation guidelines such as those from the WHO [22] recommend assessing through scaled-down validation studies and *in vitro* infectivity models, preferably using relevant viruses known to contaminate the material to be tested. Well-established *in vitro* infectivity models using such viruses have been developed for HIV, and more recently HAV and B19 virus [22]. When no *in vitro* infectivity assay exists for a relevant virus, model viruses may be used. For HCV, previous testing relied on model surrogate viruses including BVDV or Sindbis virus [22,39], for which reliable *in vitro* cell infectivity models exist.

Significant progress over the past decade has made *in vitro* culture of HCV possible, using recombinant full length genomes to produce infectious HCVcc particles in hepatoma cells [17–19]. In the advent of such development, we used a reporter-tagged HCVcc system and demonstrated the capacity of the 1% TnBP / 1% Triton X-45 SD treatment to inactivate plasma spiked with the virus by confirmation of reporter signals, virus titer, and viral NS5A protein expression levels. Our results suggest that the HCVcc *in vitro* infectivity model could serve as an important tool for evaluating viral inactivation methods in blood products. Similar observations were made recently in other pathogen reduction methods for HCV in plasma and platelet concentrates [41].

Risk of HCV transmission via transfusion of blood products remains high in resource-constrained countries and endemic parts of the world. Our data indicated that SD minipool TnBP/Triton X-45 treatment followed by C18 filtration effectively removed HCV infectivity from virus-spiked plasma without compromising the plasma hemostatic function. The propensity of SD treatment to reduce lipid molecules (Table 1) and its ability to dissolve lipid membrane likely facilitated inactivation of the enveloped HCV particles, which are highly lipidic in nature and are known to be associated with lipoproteins rich in triglyceride and cholesteryl esters [42]. The Sep-Pak Plus C18 cartridge was also observed to be efficient in removing SD-associated toxicity. In addition, such an inactivation method was highly effective against patient-derived HCV isolates and could also efficiently abrogate HCVcc infection of primary human hepatocytes. As recommended in international guidelines [22], we verified that the conditions used for this experimental scaled-down HCV inactivation study did not alter the hemostatic properties of plasma. This allowed us to confirm that the scaling-down process was conducted properly to mimic larger scale operation. PT and APTT were found to be within normal range, indicating a good preservation of plasma coagulation factors, including fibrinogen, factor VIII, and factor IX. The PT and APTT values obtained here were also close to, or marginally better, than those published previously [15,16]. This is most likely because the duration of treatment in our investigation was intentionally restricted to 30 min to evaluate the capacity to inactivate HCV, whereas previous studies were performed over 120 min as they intended to study the impact of the TnBP/Triton X-45 treatment on plasma proteins activity and plasma hemostatic activity [15,16]. As can be expected, a longer SD treatment may slightly affect the activity of coagulation factors and therefore prolong the coagulation time measured by PT and APTT. The above results therefore demonstrate the safety and clinical value of SD/C18 treatment for removal of HCV in plasma. Our study also confirms previous findings that this TnBP/Triton X-45 treatment has the capacity to quickly inactivate a high viral load of enveloped viruses as reported recently using Dengue virus [43].

In conclusion, our data using the cell culture-derived HCV infectivity model demonstrate that the minipool TnBP/Triton X-45 treatment can inactivate HCV present in the plasma, and confirm the SD procedure as a potent method for reducing plasma transfusion-associated HCV infection.
